# The Role for the Small Cryptic Plasmids As Moldable Vectors for Genetic Innovation in *Aeromonas salmonicida* subsp. *salmonicida*

**DOI:** 10.3389/fgene.2017.00211

**Published:** 2017-12-15

**Authors:** Sabrina A. Attéré, Antony T. Vincent, Mégane Paccaud, Michel Frenette, Steve J. Charette

**Affiliations:** ^1^Département de Biochimie, de Microbiologie et de Bio-informatique, Faculté des Sciences et de Génie, Université Laval, Quebec City, QC, Canada; ^2^Institut de Biologie Intégrative et des Systèmes, Université Laval, Quebec City, QC, Canada; ^3^Centre de Recherche de l’Institut Universitaire de Cardiologie et de Pneumologie de Québec, Quebec City, QC, Canada; ^4^Groupe de Recherche en Écologie Buccale, Faculté de Médecine Dentaire, Université Laval, Quebec City, QC, Canada

**Keywords:** *Aeromonas salmonicida* subsp. *salmonicida*, cryptic plasmid, Tn*1721*, pAsa10, pAsaXI, pAsaXII, antibiotic resistance

## Abstract

In *Aeromonas salmonicida* subsp. *salmonicida*, a bacterium that causes fish disease, there are two types of small plasmids (<15 kbp): plasmids without known function, called cryptic plasmids, and plasmids that bear beneficial genes for the bacterium. Four among them are frequently detected in strains of *A. salmonicida* subsp. *salmonicida*: pAsa1, pAsa2, pAsa3, and pAsal1. The latter harbors a gene which codes for an effector of the type three secretion system, while the three others are cryptic. It is currently unclear why these cryptic plasmids are so highly conserved throughout strains of *A. salmonicida* subsp. *salmonicida*. In this study, three small plasmids, named pAsa10, pAsaXI and pAsaXII, are described. Linked to tetracycline resistance, a partial Tn*1721* occupies half of pAsa10. A whole Tn*1721* is also present in pAsa8, another plasmid previously described in *A. salmonicida* subsp. *salmonicida*. The backbone of pAsa10 has no relation with other plasmids described in this bacterium. However, the pAsaXI and pAsaXII plasmids are derivatives of cryptic plasmids pAsa3 and pAsa2, respectively. pAsaXI is identical to pAsa3, but bears a transposon with a gene that encodes for a putative virulence factor. pAsaXII, also found in *Aeromonas bivalvium*, has a 95% nucleotide identity with the backbone of pAsa2. Like pAsa7, another pAsa2-like plasmid recently described, *orf2* and *orf3* are missing and are replaced in pAsaXII by genes that encode a formaldehyde detoxification system. These new observations suggest that transposons and particularly Tn*1721* are frequent and diversified in *A. salmonicida* subsp. *salmonicida*. Moreover, the discovery of pAsaXI and pAsaXII expands the group of small plasmids that are derived from cryptic plasmids and have a function. Although their precise roles remain to be determined, the present study shows that cryptic plasmids could serve as moldable vectors to acquire mobile elements such as transposons. Consequently, they could act as key agents of the diversification of virulence and adaptive traits of *Aeromonas salmonicida* subsp. *salmonicida.*

## Introduction

The *Aeromonas salmonicida* subsp. *salmonicida* bacterium is a fish pathogen which causes furunculosis to salmonids, particularly in fish farms. In this microorganism, plasmids are present in a high abundance and diversity (Dallaire-Dufresne et al., 2014b; Piotrowska and Popowska, 2015). Plasmids are self-replicative, generally circular, and extra-chromosomal DNA molecules (Lederberg, 1952). They usually bear genes that are non-essential for their hosts, but can also bring non-negligible advantages such as antibiotic resistance or virulence factors.

The classical set of plasmids found in *A. salmonicida* subsp. *salmonicida* includes the small plasmids pAsa1, pAsa2, pAsa3, and pAsal1 (5–6 kb) in addition to the large plasmid pAsa5 (155 kb) (Belland and Trust, 1989; Boyd et al., 2003; Reith et al., 2008). The plasmids pAsa1, pAsa2, and pAsa3 possess only the genes required for their replication and mobilization. Given that no function has been assigned to them yet, these plasmids are called “cryptic.” pAsa1 and pAsa3 also have genes involved in their stability since they harbor a toxin-antitoxin (TA) system (Boyd et al., 2003). TA systems are involved in plasmid stability in their host. They are composed of two molecules: a toxin and an antitoxin. The toxin is a generally stable protein, while the antitoxin that controls the activity of the toxin can be either a protein or a non-coding RNA, and is labile (Schuster and Bertram, 2013). This “addiction” system ensures that bacterial cells that did not integrate the plasmid with the TA system are killed by the remaining toxin. For the others, the antitoxin neutralizes the action of the toxin, guaranteeing the presence of the plasmid to the following generations. In pAsa1 and pAsa3, the RelEB system is the one present in those plasmids (Boyd et al., 2003); *relE* and *relB* encode for the toxin and the antitoxin, respectively (Gotfredsen and Gerdes, 1998). The fourth small plasmid, pAsal1, bears the gene *aopP*, which encodes an effector of the type three secretion system (TTSS), an important virulence factor for the bacterium (Boyd et al., 2003; Dacanay et al., 2006; Fehr et al., 2006). The large plasmid pAsa5 completes the classical set of plasmids. It supports most of the genes required to encode a functional TTSS, except for some genes located on the chromosome and pAsal1 that encode effectors (Stuber et al., 2003; Reith et al., 2008; Vanden Bergh and Frey, 2014).

Outside these plasmids, many *A. salmonicida* subsp. *salmonicida* isolates were shown to possess additional plasmids (Vincent et al., 2014, 2016; Attéré et al., 2015; Tanaka et al., 2016; Trudel et al., 2016) while others lack some of the typical ones (Boyd et al., 2003; Najimi et al., 2008; Attéré et al., 2015). For example, pAsa3 and pAsal1 are often absent in *A. salmonicida* subsp. *salmonicida* isolates, with a greater propensity to be missing in European isolates compared to the Canadian ones (Attéré et al., 2015). Interestingly, the pAsal1 plasmid shares about 70% of nucleotide identity with pAsa3, which raises the possibility that one derives from the other (Boyd et al., 2003). Another small plasmid, pAsa7, was listed in a *A. salmonicida* subsp. *salmonicida* strain from Switzerland. This plasmid is putatively a modified-pAsa2 where the *orf2* and *orf3*, which encode hypothetical proteins, were replaced by a functional gene *cat*, encoding a chloramphenicol acetyltransferase (Vincent et al., 2016).

Based on the examples of pAsal1 and pAsa7, which may be derived from pAsa3 and pAsa2 respectively, it is tempting to propose that cryptic plasmids may act as genetic innovation vectors. To further explore this proposition, we analyzed a set of 22 *A. salmonicida* subsp. *salmonicida* isolates and identified one new non-typical small plasmid. This new small replicon, and two others previously found but not described (Trudel et al., 2013; Attéré et al., 2015), were sequenced and characterized. In addition to serve as gene shuttles for bacteria that share the same environment, two of them add evidence that small cryptic plasmids might have a major evolutionary role: acting as moldable vectors to spread new genes through lateral gene transfers.

## Materials and Methods

### Growth of Bacterial Strains

When required, the 22 strains of *A. salmonicida* subsp. *salmonicida* listed in Supplementary Table 1 were streaked on furunculosis agar medium (10 g of bacto tryptone, 5 g of yeast extract, 2.5 g of sodium chloride, 1 g of L-tyrosine, 15 g of agar per liter of water), and then incubated at 18°C for 48 h. Colonies were then used either for plasmid extraction or lysate production for PCR genotyping. Strains 01-B526, JF3791 and HER1084 were also streaked in the same conditions before the determination of the minimum inhibitory concentration for formaldehyde. Strains 2004-208 and HER1084 were already partially analyzed in previous studies (Trudel et al., 2013; Attéré et al., 2015) and were investigated further in the present one.

### Plasmid Profile

Plasmid extraction of every strain was made with the QIAprep Spin Miniprep Kit (QIAGEN). Then the restriction enzyme *Eco*RI was used to digest all plasmids (Boyd et al., 2003) with the following conditions: 25 μL of the preparation digested with the *Eco*RI-HF^®^ of New England BioLabs (NEB) for 2 h at 37°C. DNA digestions were migrated on a 0.7%-agarose gel for 75 min at 90 V and the plasmid profiles were visualized with ethidium bromide under UV.

### PCR for Small Plasmids and Antibiotic Resistance

PCR genotyping was also used for the detection of the standard small plasmids (pAsa1, pAsa2, pAsa3, and pAsal1). DNA lysates were obtained as follows: after resuspension of bacterial colonies in water, they were centrifuged for 5 min, at 3 385 × *g*. Then the supernatant was replaced by lysis buffer SWL (0.186 g of potassium chloride, 0.061 g of Tris-Base, 0.254 g of magnesium chloride, 225 μL of Tween 20 and NP40, per 50 mL of water). The whole was then incubated at 95°C for 15 min. Lysates were then diluted to a DNA concentration of 100 ng/μL. The conditions and the primers of the PCR used to highlight all the standard small plasmids of these strains from the DNA lysates were previously described (Attéré et al., 2015). Genes for chloramphenicol, florfenicol, sulfonamides and tetracycline resistance were detected with a previously described multiplex PCR approach (Trudel et al., 2016).

### Determination of the Minimum Inhibitory Concentration for Formaldehyde

Resistance to formaldehyde was evaluated for HER1084 (pAsaXII), 01-B526 (standard plasmidome) and JF3791 (pAsa7, derived from pAsa2). Strains 01-B526 and JF3791 have been used as negative controls since they do not possess any known formaldehyde detoxification system. The three strains were cultured in liquid LB medium overnight and the optical density (OD_595_) adjusted to 0.2. For each strain, volumes of 100 μL were distributed in a 96-well microplate containing formaldehyde that reached final concentrations ranging from 0.0031 to 1% (serially diluted) to determine firstly the rank of the resistance, then from 0.00031 to 0.01% (serially diluted) for a total volume of 200 μL by well. The OD_595_ was then taken every 15 min for 48 h at 18°C in a TECAN Infinite Pro (Tecan, Morrisville, NC, United States). The second experiment has been done in triplicate.

### Plasmid Sequencing and Bioinformatics Analyses

High-throughput sequencing of the small plasmid DNA by Illumina MiSeq technology (2 × 300 bp) was performed on the mini-preparation DNA extraction of strains SHY15-2743, 2004-208 and HER1084. The reads generated were then randomly subsampled using seqtk^1^ and *de novo* assembled with A5-miseq version 20160825 (Coil et al., 2015). Assembly information is provided (Supplementary Table 2). The plasmid sequences were circularized using various tools included in the package EMBOSS version 6.6.0.0 (Rice et al., 2000). The annotations of the plasmids were done automatically with RAST (Aziz et al., 2008), and further manually curated. TAfinder 2.0 was used to predict toxin and antitoxin genes (Shao et al., 2011). All the sequences were visualized and annotated with Artemis software 16.0.0 (Rutherford et al., 2000). The sequences of pAsa10, pAsaXI and pAsaXII were deposited in the DDBJ/ENA/GenBank databases under the accession numbers MF621616, MF621617 and MF621618, respectively. Easyfig 2.1 was used to visualize the comparisons between sequences (Sullivan et al., 2011).

## Results and Discussion

### Plasmid Repertoire

**FIGURE 1:**
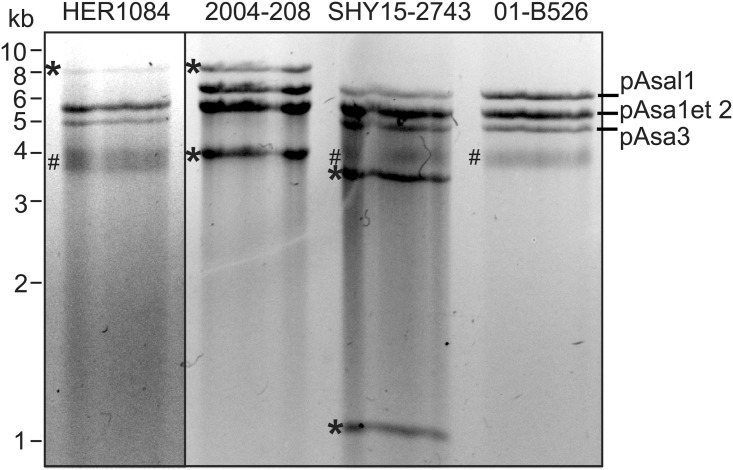
Atypical small plasmid profiles of strains HER1084, 2004-208 and SHY15-2743. A small plasmid extraction of each strain was digested with *Eco*RI prior to agarose gel electrophoresis. Each strain bears one or two additional bands (marked with ^∗^). Strain 01-B526 harbors a typical plasmid profile composed of the non-cryptic pAsal1 plasmid, and pAsa1, pAsa2, and pAsa3 plasmids with unknown functions. Incompletely digested plasmidic DNA was also present (#).

Plasmid profiling of 22 strains of *A. salmonicida* subsp. *salmonicida* revealed that all of them possessed the small cryptic plasmids pAsa1, pAsa2 and pAsa3 (Supplementary Table 1). Among these strains, six did not have plasmid pAsal1. This observation is in accordance with another study, which reported that plasmid pAsal1 is unstable, likely due to the lack of a “cell addiction” system, such as a toxin/antitoxin system (Attéré et al., 2015). Only strain SHY15-2743 harbored an atypical plasmid profile, which suggested the presence of a new plasmid (**Figure 1**). This strain was also shown by PCR assay to possess a *tetA* gene, involved in tetracycline resistance (Supplementary Table 1).

Previously, two studies allowed us to discover that strains 2004-208 and HER1084 possessed atypical plasmidic profiles (Trudel et al., 2013; Attéré et al., 2015). The small plasmid profiles obtained after *Eco*RI digestion of these two strains (2004-208 and HER1084) (**Figure 1**) showed that strain HER1084 did not have the pAsal1 plasmid, but presented an 8-kb band. With the exception of pAsa3, all small usual plasmids were found in strain 2004-208. Its atypical profile also includes two supplementary bands at about 4 and 8 kb. For SHY15-2743, there were two additional bands compared to the standard plasmid profile: one at 3.5 kb and another at 1 kb.

To shed light on the molecular and evolutionary history of these new plasmids, their DNA were sequenced using a high-throughput technology. Strains SHY15-2743, 2004-208 and HER1084 were shown to possess one new plasmid each: pAsa10, pAsaXI and pAsaXII, respectively. Here, we propose a new nomenclature for *A. salmonicida* plasmids. All plasmids after pAsa10 should be named in Roman numerals to avoid any confusion due to fonts, for example pAsal1 and pAsa11 (pAsaXI).

### pAsa10

**FIGURE 2:**
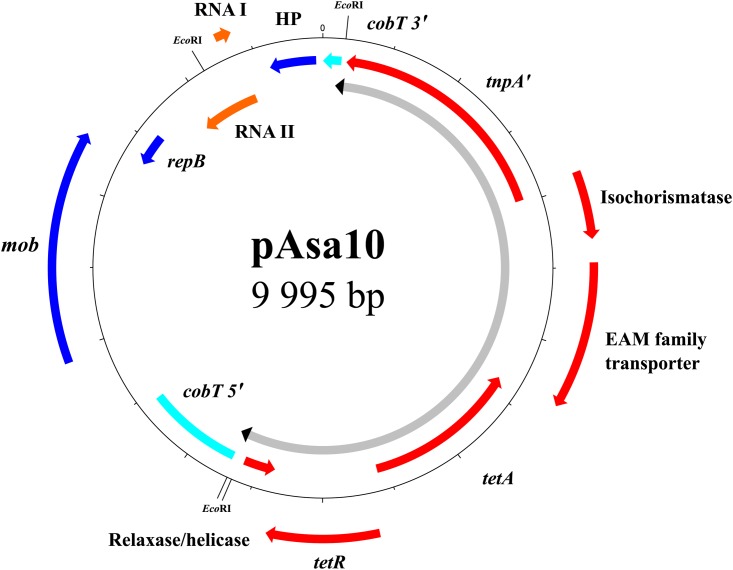
Map of the pAsa10 plasmid found in strain SHY15-2743. The tetracycline resistance is borne by the *tet* region of transposon Tn*1721*. Tn*1721* interrupts gene *cobT* involved in the biosynthesis of cobalamin. The genes of the mobile genetic element are shown in red, the pseudogene in turquoise, the genes of the backbone in blue, and the RNAs in orange. The portion of the plasmid comprising the transposon is shown in gray with the inverted repeats in black.

In strain SHY15-2743, the presence of a new 10-kb plasmid named pAsa10 made the link between the detected tetracycline resistance gene *tetA* and the atypical plasmid profile (Supplementary Table 1). This plasmid increases the number of those allowing resistance to this antibiotic in *A. salmonicida* subsp. *salmonicida* (L’Abee-Lund and Sorum, 2002; Vincent et al., 2014; Tanaka et al., 2016; Trudel et al., 2016). Effectively, bioinformatics analyses and multiplex PCR revealed that the genes *tetA* and *tetR* involved in tetracycline resistance are present on pAsa10, and caused by a partial transposon *1721* (Tn*1721*) (**Figure 2**).

Tn*1721* is a mobile genetic element discovered for the first time in the plasmid pRSD1 of *Escherichia coli* D1021 (Burkardt et al., 1978; Schmitt et al., 1979). This transposon is composed of two parts (Allmeier et al., 1992). Its precise mechanism of formation is still unknown but it is accepted that two transposons Tn*1722* were initially involved in this process. More precisely, these transposons were inserted on both sides of a *tet* region and a part of one of them was deleted to give the actual Tn*1721*. So, Tn*1721* consists of a whole Tn*1722* named “minor transposon” and a *tet* region comprising the genes *tetA*, *tetR* with the truncated transposase of the other Tn*1722* (Schmitt et al., 1981). Transposon Tn*1721* is also characterized by the presence of three 38-pb inverted repeats (IRs) and two 5-bp direct repeats (DRs) due to the insertion of the transposon (Schoffl et al., 1981).

**FIGURE 3:**
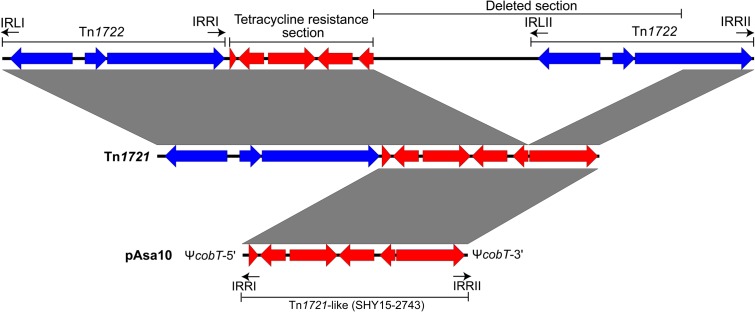
Formation of transposon Tn*1721* and atypical orientation of its IRs in the pAsa10 plasmid. The formation of Tn*1721* is the result of a partial deletion of transposon Tn*1722*, localized near another Tn*1722*, itself next to genes responsible for tetracycline resistance (Schmitt et al., 1981). The *tet* region is in red and the complete transposon Tn*1722* in blue.

Two identical IRs, IRRI and IRRII, surround the *tet* region in the same orientation while the third one, IRL, is in an opposed orientation compared to the others, and near to the “minor transposon” (**Figure 3**) (Schmitt et al., 1979). This IR has mutations that differentiate it from the previous two. Consequently, Tn*1721* could transpose in two ways; either all the mobile genetic element can spread from one site to another or only the “minor transposon Tn*1722*” is transferred (Schoffl et al., 1981).

The *tet* region of Tn*1721* is present in the pAsa10 plasmid (**Figure 2**). This section of the transposon interrupts *cobT*, a gene involved in the biosynthesis of cobalamin (Cameron et al., 1991; Trzebiatowski et al., 1994), and for which the coding DNA sequence was reconstituted *in silico* to assess it completeness. This is the first time that a gene implicated in the biosynthesis of this product is discovered on a plasmid in *A. salmonicida* subsp. *salmonicida*. The insertion of the partial Tn*1721* resulted, from either side, in the appearance of the two 5-bp DRs, with the palindromic sequence 5′-GTCTG-3′. However, the IRRI is in a reverse orientation comparatively to the one found in the reference Tn*1721* (**Figure 3**). The mechanism to explain this inversion remains to be determined, but it is likely that this could be linked with the fact that the transposon is only partial in pAsa10. In any case and to the best of our knowledge, this is the first time that IRRI and IRRII are reported in an opposite direction in a partial Tn*1721*.

**FIGURE 4:**
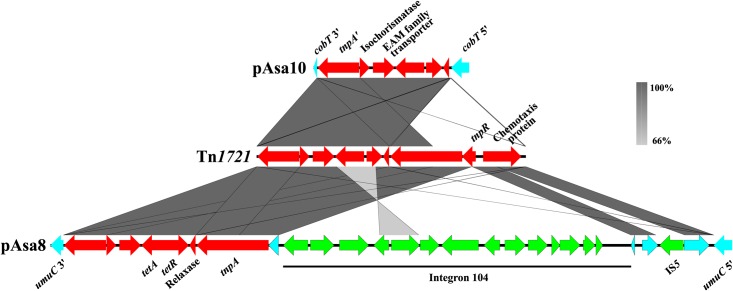
Comparison of the known and sequenced Tn*1721* in plasmids of *A. salmonicida* subsp. *salmonicida.* The gray zones represent similarities between pAsa10, Tn*1721* and pAsa8 (accession numbers MF621616, X61367 and KX364409, respectively). The genes of Tn*1721* are shown in red, the pseudogene in turquoise, and the other mobile genetic elements in green.

Interest for Tn*1721* is also increasing because of its spread in the plasmidome of *A. salmonicida* subsp. *salmonicida*. **Figure 4** shows a comparison of the known and sequenced Tn*1721* in this bacterium. A previous study revealed its presence in the pAsa8 plasmid (Trudel et al., 2016). In this case, the interrupted gene and the DR sequences were different from those of pAsa10. Moreover, Tn*1721* was complete, but its sequence was cut by two other mobile genetic elements: one insertion sequence (IS*5*) found in the gene encoding a chemotaxis protein, and an integron (In104) bearing two cassettes with several genes of antibiotic resistance. Acquisition of this transposon, and its potential ability to integrate other mobile elements, could represent a new way for *A. salmonicida* subsp. *salmonicida* to recombine, disrupt genes, and integrate other mobile elements, could not only represent a new way for *A. salmonicida* subsp. *salmonicida* to circumvent the antibiotic treatments pressure, but also to adapt to new environment conditions.

### pAsaXI

**FIGURE 5:**
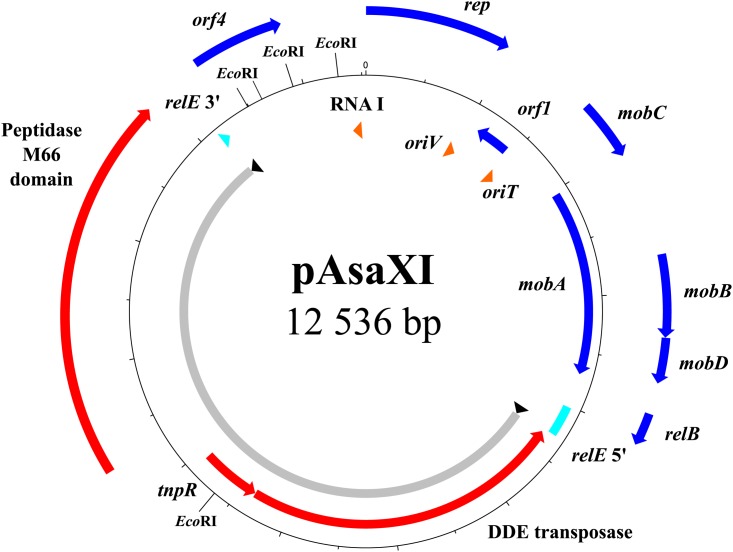
Map of the pAsaXI plasmid. The genes of the mobile genetic element are shown in red, the pseudogene in turquoise, the genes of the backbone in blue, and the RNA with the *oriT* and *oriV* in orange. The portion of the plasmid comprising the transposon is shown in gray with the inverted repeats in black.

Another new plasmid characterized here is pAsaXI. Firstly, this plasmid brings a potential virulence factor to the carrier strain 2004-208 (**Figure 5**). Indeed, pAsaXI possesses a 6.9-kb transposon composed of three genes: one that encodes a DDE transposase that belongs to the Tn3-family according to a BLASTp analysis, the *tnpR* gene, and a gene that encodes a protein having a peptidase M66 domain (positions 236–537, *e*-value of 1.57e^-89^) preceded by a signal peptide. A BLASTp analysis revealed that the sequence of the latter gene is likely an homolog of *stcE* found in *E. coli* (71% of identity over 99% of the query length) known to be a sequelog of *tagA* found in *Vibrio cholerae* and *Aeromonas hydrophila*, which are involved in the virulence of these strains (Lathem et al., 2002; Varshavsky, 2004; Pillai et al., 2006; Szabady et al., 2011). Secondly, like other transposons, this one is surrounded by two IRs, and generated 5-pb DRs with the palindromic sequence 5′-ATATA-3′. Except for the presence of the transposase, the length and the sequences of the IRs differ from those found in transposons of Tn3 family (Siguier et al., 2015). Indeed, IRs are shorter (32 pb), and, even if they begin with the conserved “GGGG” motif, the end of their sequences does not comprise the final TAAG (Siguier et al., 2015). Consequently, we can infer that this transposon belongs likely to a Tn3-family like, but to date, this transposon is mentioned nowhere in the literature. However, a BLAST analysis revealed that the whole transposon is also present in three uncharacterized plasmids of the bacterium *Shewanella baltica* (GenBank # CP002384.1, CP001255.1 and CP001253.1). The latter, previously named *Shewanella putrefaciens*, colonizes fresh and marine water, and was isolated from the Baltic Sea (Ziemke et al., 1998). Consequently, finding this transposon in a strain of *A. salmonicida* subsp. *salmonicida* suggests that at least one direct or indirect event of horizontal transfer involving these bacteria occurred, and demonstrates one more time the high capacity of *A. salmonicida* subsp. *salmonicida* to exchange DNA with other species.

Thirdly, the pAsaXI plasmid is a variant of pAsa3, since its backbone sequence is the same as the one of the reference strain A449 (GenBank: NC_004924). Interestingly, it is likely that pAsa3 is absent in 2004-208, or present in a small subpopulation of cells, for at least two reasons: firstly, it was not viewable by the gel electrophoresis (**Figure 1**), and secondly, investigation of the sequencing reads that overlap the region in *relE* where the transposon has been inserted revealed that more than 99% of these reads correspond to the transposon. This observation suggests that plasmid pAsaXI has taken advantage over plasmid pAsa3 in strain 2004-208. The discovery of pAsaXI is a new proof that might lead us to consider pAsal1 as a real derivative of pAsa3, and no longer as a putative variant of pAsa3 (see Cryptic Plasmids: A Way for Genetic Innovation in *A. salmonicida* subsp. *salmonicida*?).

In bacteria, the TA systems are involved in plasmid stability. Generally, the toxin, more stable than the antitoxin, ensures plasmid maintenance by killing cells in which the plasmid is lost (Jensen and Gerdes, 1995). The genes are frequently organized such that the gene of the antitoxin is upstream from the gene of the toxin. In pAsaXI, the transposon interrupts *relE*, which encodes the toxin of the TA *relBE* system (Gotfredsen and Gerdes, 1998). This system is likely involved in the stability of pAsa1 and pAsa3 plasmids in *A. salmonicida* subsp. *salmonicida* (Boyd et al., 2003). Therefore, we can infer that the *relBE* system had to fulfill the same function in pAsaXI. However, the insertion in *relE* leads us to consider that this precise place could be a way to the bacterium (1) to protect itself against this toxic system, and (2) in the same time, to gain beneficial functions; and for the plasmid which is compact, it is likely a place where newly acquired genes could be maintained without affecting the replication and the mobilization of the plasmid.

This strategy of getting rid of the toxin, while keeping the antitoxin, could prevent cell death. Effectively, in the case that the cells become confronted again to the toxin of pAsa3, for example by reacquiring it by a horizontal transfer event, the antitoxin of pAsaXI would likely be able to play its protective role. Moreover, the putative virulence factor that the transposon carries lends another advantage to the bacterium, and could be pressure for the plasmid to be maintained in cells. The localization of the transposon suggests that this TA system could be lost without affecting the plasmid maintenance, and that another mechanism is likely involved to ensure the stability of pAsaXI. We could not exclude the possibility that this plasmid is in high copy number in the cells, since it is a derivative of the ColE2-type replicon pAsa3, and that would be enough to ensure the transmission to daughter cells.

### pAsaXII

**FIGURE 6:**
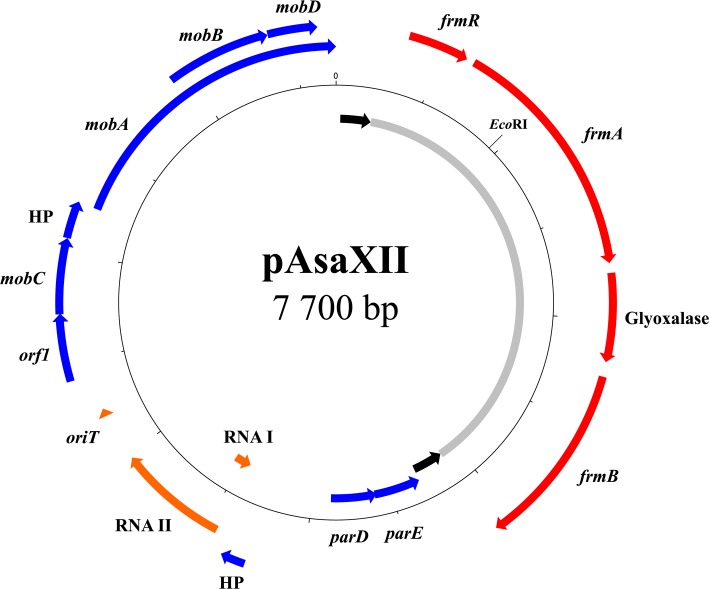
Map of pAsaXII plasmid. Almost half of plasmid pAsaXII is occupied by a formaldehyde detoxification system (gray zone) flanked by two IRs (black arrows). Like pAsaXI, a MIC-like element interrupts the STOP codon of the gene *parE* encoding a toxin. The genes of the mobile genetic element are shown in red, the pseudogene in turquoise, the genes of the backbone in blue, and the RNAs and *oriT* in orange.

The last discovered plasmid in this study is pAsaXII, found in strain HER1084, isolated from a fish captured in France (**Figure 6**). As with pAsaXI, this third plasmid is a derivative of a cryptic plasmid (pAsa2 in this case) and contains a putative mobile insertion cassette (MIC) that interrupts, here again, a TA system. More precisely, the MIC is inserted in the *parE* gene, which encodes for the toxin of the *parED* TA system (Roberts et al., 1994). Like pAsa7 (Vincent et al., 2016), pAsaXII highlights that cryptic plasmid pAsa2 could be modified, and form new variants (see Cryptic Plasmids: A Way for Genetic Innovation in *A. salmonicida* subsp. *salmonicida*?). Interestingly, pAsa2 was detected by gel electrophoresis and bioinformatics analyses. Moreover, the regulator RNAs between pAsa2 and pAsaXII exhibit several differences that likely allow both plasmids to coexist in the same cells, as required for ColE1-type replicon plasmids (Camps, 2010; Vincent et al., 2016).

The presence of pAsaXII in *A. salmonicida* subsp. *salmonicida* illustrates again the strong ability of this bacterium to acquire or transfer foreign DNA. Effectively, although unlisted in the scientific literature, a BLASTn analysis allowed us to find the same plasmid in *Aeromonas bivalvium* (Genbank accession number CDBT01000031). The fact that *A. salmonicida* subsp. *salmonicida* is known to be psychrophilic while *A. bivalvium* is mesophilic (Minana-Galbis et al., 2007) introduces the possibility of genetic transfers between aeromonads of different lifestyles.

This transfer demonstrates that functions other than antibiotic resistance can be acquired in *A. salmonicida*, enlarging its pan-genome and potentially increases its adaptation to different environments. Indeed, pAsaXII also bears four genes involved in a detoxification system for formaldehyde. Bacteria can possess resistance to this molecule, and several pathways for formaldehyde detoxification have been discovered (Chen et al., 2016). In *E. coli*, one of these pathways (the GSH-dependent pathway) involves genes of the operon *fmrAB* (Herring and Blattner, 2004; Chen et al., 2016), and these are present on plasmid pAsaXII. However, the organization of this operon is slightly different in pAsaXII since a gene that encodes a glyoxalase separates genes *fmrA* and *fmrB*.

This detoxification system, reported for the first time in *A. salmonicida* subsp *salmonicida*, has been inserted in a putative MIC (Siguier et al., 2015). Indeed, the operon *fmrAB* is flanked by two IRs that are quasi-identical (96% identity), and direct repeats could be detected (5′-TAAA-3′). However, the DR sequences are not directly after the IRs, as usually found in transposons; one different nucleotide (G and C) separates them, from either side of the putative MIC. The reason for this unusual feature is still unknown and may simply be caused by an erosion of the sequence due to the lack of conservative pressure. Finally, there is no gene that encodes a transposase, which suggests that the gene was lost or provided in *trans*. Concerning the functionality of the operon, we determined that the strain HER1084 has a minimum inhibitory concentration for formaldehyde of 0.01% comparatively to 0.0025% for the strains 01-B526 and JF3791. This result suggests that the detoxification system encoded by the operon *fmrAB* on pAsaXII is functional. Since strains 01-B526 (standard repertoire of plasmids) and JF3791 (which possesses pAsa7, a derivative of pAsa2) are more sensitive than strain HER1084 (which bears pAsaXII), it is possible to consider that standard plasmids (pAsa1, pAsa2, pAsa3, pAsal1, and pAsa5) or pAsa7 do not confer resistance to formaldehyde even if it is impossible to rule out other strain-specific characteristics.

Use of formaldehyde as a disinfectant for surfaces goes back at least at the end of the 19th century (Harrington, 1897). In aquaculture, it has been employed to treat or prevent fungal infections, and external parasites of fish or their eggs (Bills et al., 1977). It has also been widely used to treat fish before their introduction into a new farm. As for antibiotics, resistance genes against this molecule exist, and as described in this study can be transferred with plasmids and/or other mobile genetic elements. Consequently, even if, to our knowledge, formaldehyde has not been used to cure furunculosis, the bacterium already possesses a mechanism to resist, likely caused by the years of formaldehyde’s employment in prophylactic treatments.

### Cryptic Plasmids: A Way for Genetic Innovation in *A. salmonicida* subsp. *salmonicida*?

As previously described (Attéré et al., 2015), the presence of the cryptic plasmids and their potential modifications (SNPs and InDels) were investigated in the strains bearing the new plasmids (Supplementary Table 3). Interestingly, identical mutations to those found before have been identified (Attéré et al., 2015). pAsa2 in strain HER1084 (pAsaXII carrier) has been modified, as the one in strain RS1752, and presents a duplication of 43 bp in an intergenic region. Moreover, analyses suggest that this region could form stem loop structures, which are characteristic of regulatory regions (Supplementary Figure 1). Since this strain bears pAsaXII, variant of pAsa2, it would be possible that it is a way for the plasmid to be maintained in the bacterium. Concerning pAsa3, this plasmid remains the one in which there are the most mutations.

Since their discovery, cryptic plasmids are known as plasmids without assigned functions (Boyd et al., 2003). pAsa1, pAsa2, and pAsa3 only bear the genes necessary for their mobilization, replication, and stability. Some ORFs are still not characterized, but since the number of sequenced and available genomes is increasing in many databases, indications can be found about possible domains that are present on the proteins encoded by *orf*2 and *orf*3 of plasmid pAsa2. Indeed, the protein obtained from *orf2* would have a RES domain hence the name come from three residues (arginine, glutamate and serine) that would be involved in the formation of an active site (pfam: 08808). A helix-turn-helix domain would be found on the protein encoded by the gene *orf3*. This domain suggests the ability of this protein to bind DNA (pfam: 127280). For *orf1* and *orf4*, to date, no information of this type can be found. But the presence of *orf1* on plasmids of many bacterial species (based on BLAST analyses) may lead us to think that this is a conserved gene, which probably has an important role for the plasmids. In any case, the lack of information about these ORFs may contribute to our partial comprehension of the real roles of cryptic plasmids, and subsequent investigations should be conducted to determine the precise functions of each of these ORFs.

**FIGURE 7:**
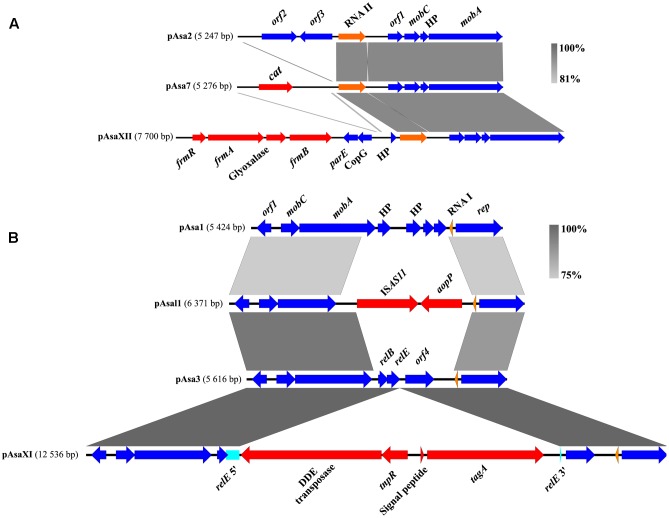
Sequence comparisons of some ColE1- and ColE2-plasmids of *A. salmonicida*. **(A)** In the case of pAsa2 and its derivatives pAsa7 and pAsaXII (ColE1 plasmids), the region of *orf2* and *orf3* are substituted by a chloramphenicol resistance gene or a formaldehyde detoxification system, respectively. **(B)** For pAsa3 (ColE2), genes *relE*, *relB* and *orf4* are included in the region that can likely be modified or replaced. The genes of the mobile genetic element are shown in red, the pseudogene in turquoise, the genes of the backbone in blue, and the RNAs in orange.

Nevertheless, the pAsaXI and pAsaXII plasmids described in this study present new clues about the potential functions of cryptic plasmids, and for pAsa2, about *orf2* and *orf3*. Since these new plasmids share backbones with pAsa3 and pAsa2 respectively, we compared them with the “original” cryptic plasmids and their variants (**Figure 7**). For the ColE1-type replicon plasmids (pAsa2 and its variants pAsa7 and pAsaXII) the region including *orf2* and *orf3* is lost in the derivatives of pAsa2. Indeed, this lost part has been replaced by a DNA segment including the gene *cat* for pAsa7 (Vincent et al., 2016), and by the putative MIC bearing the detoxification system for formaldehyde in pAsaXII. This observation suggests that *orf2* and *orf3* are non-essential for pAsa7 and pAsaXII plasmids, and/or that the proteins produced by these ORFs are brought in *trans* by plasmid pAsa2, since it is present in the strains that bear pAsa7 and pAsaXII.

For ColE2-plasmids (pAsa1, pAsa3, pAsal1 and pAsaXI), the comparison permitted the same conclusion as for ColE1-plasmids: a specific region tends to be lost or modified easily. Effectively, the *relEB* TA system and *orf4* are replaced in pAsal1 by the IS*AS11* and gene *aopP* while in pAsaXI, gene *relE* is interrupted by a transposon that bears a virulence factor (**Figure 7**).

The replicon of pAsa3 had already been used to create a vector, pSDD1, to perform molecular biology assays in *A. salmonicida* subsp. *salmonicida* (Dallaire-Dufresne et al., 2014a). This ColE1-type vector was shown to be unstable and is lost when bacterial strains that carry it are cultured for 24 h without selection (without ampicillin). Knowing that pAsaXI and pAsaXII are derivatives of small cryptic plasmids, like pSDD1, it was relevant to verify their stability. Opposite to pSDD1, both pAsaXI and pAsaXII are stable in their natural hosts, even after 7 days of culture without selection. This result was expected since these plasmids were naturally found in *A. salmonicida* subsp. *salmonicida*, while pSDD1 is a laboratory-made chimeric plasmid.

Confronted with different environments, the bacterium *A. salmonicida* subsp. *salmonicida* seems to have found a way to adapt itself: using the cryptic plasmids present in its genome to acquire new beneficial genes. Considering several points, this could be a “winning” strategy for the bacterium: (1) a gain facing environmental conditions which can be lethal for the bacterium (antibiotics, disinfectant, etc.), (2) a zero or minimal requirement for any metabolism adaptation because cryptic plasmids used as moldable vectors are already borne by the microorganism, (3) stability of the plasmids in the bacterial cells provides consequently, in a certain way, a large retention of the acquired advantage, and (4) these plasmids are in high copy number and consequently so are the genes they bear (Vincent et al., 2016).

## Conclusion

The highlighting of the three new plasmids pAsa10, pAsaXI and pAsaXII of *A. salmonicida* subsp. *salmonicida* demonstrated once again the genome plasticity of this fish pathogen. The spread of the Tn*1721* in this bacterium, shown with pAsa10, constitutes a new way to resist tetracycline. The presence of pAsaXI and pAsaXII, variants of the small cryptic plasmids pAsa3 and pAsa2 respectively, demonstrated the high capacity of *A. salmonicida* to exchange plasmids with other species. Moreover, they showed the potential role of these cryptic plasmids as moldable vectors for acquisition of new beneficial genes, facilitating the adaptation of the carrier strains to different environments.

## Author Contributions

SA, AV, and SC designed the research protocol. SA and MP performed the experiments. SA and AV analyzed the data. SA, AV, MF, and SC wrote the paper.

## Conflict of Interest Statement

The authors declare that the research was conducted in the absence of any commercial or financial relationships that could be construed as a potential conflict of interest.

## Footnotes

1 https://github.com/lh3/seqtk
