# Supplementary material for: The Role for the Small Cryptic Plasmids As Moldable Vectors for Genetic Innovation in Aeromonas salmonicida subsp. salmonicida
Source: Front Genet. 2017 Dec 15;8:211. doi: 10.3389/fgene.2017.00211 (PMC5736529; doi:10.3389/fgene.2017.00211)
Supplement: Supplementary file 1 [file Presentation_1.PDF]

frontiers

Supplementary Material

The role for the small cryptic plasmids as moldable vectors for genetic innovation in *Aeromonas salmonicida* subsp. *salmonicida*

Sabrina A. Attéré, Antony T. Vincent, Mégane Paccaud, Michel Frenette and Steve J. Charette*

*** Correspondence:** Corresponding Author: Steve.charette@bcm.ulaval.ca

1 Supplementary Figures and Tables

1.1 Supplementary Figures

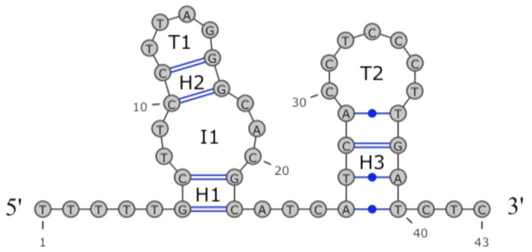

Structure 1
ΔG = -3.79 kcal/mol

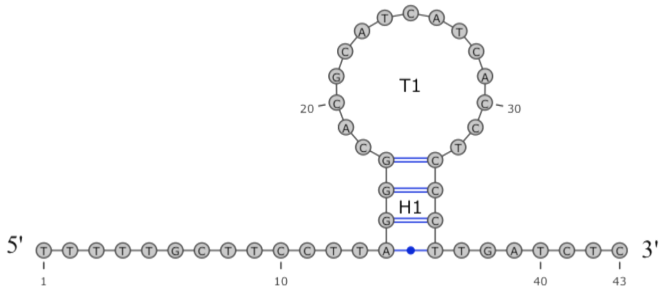

Structure 2
ΔG = -3.57 kcal/mol

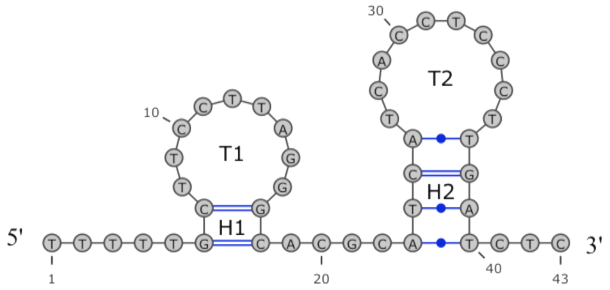

Structure 3
ΔG = -3.40 kcal/mol

Supplementary Figure 1. Predicted structures of the duplication of 43 bp in the modified-pAsa2 of strain HER1084. For this sequence located in the intergenic region of the plasmid pAsa2, three predicted structures are proposed with mfold software at 18°C (Zuker, 2003). The nomenclature used is the one of Leontis & Westhof (Leontis and Westhof, 2001). The 'H', 'I' and 'T' mean, respectively, 'Helix', 'Internal loop' and 'Terminal loop'. The change in free energy (ΔG) required for the DNA sequence to hybridize in kilocalories per mole (kcal.mol^{-1}, where 1 kcal=4184 J) is given for each structure.

Leontis, N. B., and Westhof, E. (2001). Geometric nomenclature and classification of RNA base pairs. *Rna* 7, 499–512. doi:10.1017/S1355838201002515.

Zuker, M. (2003). Mfold web server for nucleic acid folding and hybridization prediction. *Nucleic Acids Res* 31, 3406–3415. doi:10.1093/nar/gkg595.

1.2 Supplementary Tables

Supplementary Table 1: Characterization of 22 Canadian isolates of *Aeromonas salmonicida* subsp. *salmonicida* for their small plasmids and antibiotic resistance genes.

			Plasmids					Antibiotic resistance genes								
Name of the strain	Fish	Organ	pAsa				Atypical profile	*sul*		*floR*	*cat*	*tet*				
			1	2	3	l1		*1*	*2*			*A(E)*	*A*	*A(C)*	*H*	*G*
SHY15-1459	Rainbow trout	Kidney	+	+	+	-	-	-	-	-	-	-	-	-	-	-
SHY15-1543	Brook trout	Kidney	+	+	+	+	-	-	-	-	-	-	-	-	-	-
SHY15-1846	Brook trout	Kidney	+	+	+	-	-	-	-	-	-	-	-	-	-	-
SHY15-1847	Arctic char	Kidney	+	+	+	-	-	-	-	-	-	-	-	-	-	-
SHY15-2589	Arctic char	Kidney	+	+	+	+	-	-	-	-	-	-	-	-	-	-
SHY15-2743	Rainbow trout	Kidney	+	+	+	+	+	-	-	-	-	-	+	-	-	-
SHY15-1998	Brook trout	Kidney	+	+	+	+	-	-	-	-	-	-	-	-	-	-
SHY15-2405	Brook trout	Kidney	+	+	+	+	-	-	-	-	-	-	-	-	-	-
SHY15-2407	Brook trout	Kidney	+	+	+	+	-	-	-	-	-	-	-	-	-	-
SHY15-2461	N/A	N/A	+	+	+	+	-	-	-	-	-	-	-	-	-	-
SHY15-2816	Brook trout	Kidney	+	+	+	+	-	-	-	-	-	-	-	-	-	-
SHY15-2951	Brook trout	Kidney	+	+	+	+	-	-	-	-	-	-	-	-	-	-
SHY16-4166	N/A	N/A	+	+	+	-	-	-	-	-	-	-	-	-	-	-
SHY16-4688	N/A	N/A	+	+	+	+	-	-	-	-	-	-	-	-	-	-
SHY15-5108	Brook trout	Kidney	+	+	+	+	-	-	-	-	-	-	-	-	-	-
SHY15-3738	Brook trout	Kidney	+	+	+	+	-	-	-	-	-	-	-	-	-	-
SHY15-4029	Brook trout	Furuncle	+	+	+	-	-	-	-	-	-	-	-	-	-	-
SHY15-3379	Brook trout	Kidney	+	+	+	+	-	-	-	-	-	-	-	-	-	-

Name of the strain	Fish	Organ	Plasmids: pAsa 1	2	3	11	Atypical profile	Antibiotic resistance genes: *sul* *1*	*2*	*floR*	*cat*	*tet* *A(E)*	*A*	*A(C)*	*H*	*G*
SHY15-3412	Brook trout	Furuncle	+	+	+	-	-	-	-	-	-	-	-	-	-	-
SHY15-3138	Brook trout	Kidney	+	+	+	+	-	-	-	-	-	-	-	-	-	-
SHY15-3292	Brook trout	Kidney	+	+	+	+	-	-	-	-	-	-	-	-	-	-
SHY15-2950	Brook trout	Kidney	+	+	+	+	-	-	-	-	-	-	-	-	-	-

Supplementary Table 2: Assembly information about the three plasmids.

Plasmid	Strain	Length (bp)	GC (%)	Number of reads	Coverage (x)
pAsa10	SHY15 2743	9995	61	7961	148.65
pAsaXI	2004-208	12536	55	11481	171.94
pAsaXII	HER1084	7700	55	4056	108.04

Supplementary Table 3: Sequence differences (SNPs and InDels) found in the small plasmids of the three strains further analyzed in this study.

Isolate (new plasmid)	pAsa1	pAsa2	pAsa3	pAsa11
**2004-208 (pAsaXI variant of pAsa3)**				
**SHY15 2743 (pAsa10)**			Insertion G (1458)<br>C>G (2838) in *rep* and causes a (P->A)<br>C>G (2859) in *rep* and causes a (P->A)<br>G>T (3056) in HP and causes a (R->S)<br>Synonymous in *mobA*, A->C (5136) in *mobB* and causes a (E->A)	G->C (1448) in *mobA* and causes a (E->Q)<br>A->G (1450) in *mobA* and causes a (E->Q)
**HER1084 (pAsaXII variant of pAsa2)**		Extension of 43 pb	C>G (2838) in *rep* and causes a (P->A)<br>C>G (2859) in *rep* and causes a (P->A)<br>Synonymous in *mobA*, A->C (5136) in *mobB* and causes a (E->A)	

Legend:

Present and OK

Absent
